# Proof-of-Concept, Randomized, Controlled Clinical Trial of Bacillus-Calmette-Guerin for Treatment of Long-Term Type 1 Diabetes

**DOI:** 10.1371/journal.pone.0041756

**Published:** 2012-08-08

**Authors:** Denise L. Faustman, Limei Wang, Yoshiaki Okubo, Douglas Burger, Liqin Ban, Guotong Man, Hui Zheng, David Schoenfeld, Richard Pompei, Joseph Avruch, David M. Nathan

**Affiliations:** 1 The Immunobiology Laboratory, Massachusetts General Hospital and Harvard Medical School, Boston, Massachusetts, United States of America; 2 Department of Biostatistics, Massachusetts General Hospital, Boston, Massachusetts, United States of America; 3 Diabetes Unit, Massachusetts General Hospital, Boston, Massachusetts, United States of America; Statens Serum Institute, Denmark

## Abstract

**Background:**

No targeted immunotherapies reverse type 1 diabetes in humans. However, in a rodent model of type 1 diabetes, Bacillus Calmette-Guerin (BCG) reverses disease by restoring insulin secretion. Specifically, it stimulates innate immunity by inducing the host to produce tumor necrosis factor (TNF), which, in turn, kills disease-causing autoimmune cells and restores pancreatic beta-cell function through regeneration.

**Methodology/Principal Findings:**

Translating these findings to humans, we administered BCG, a generic vaccine, in a proof-of-principle, double-blind, placebo-controlled trial of adults with long-term type 1 diabetes (mean: 15.3 years) at one clinical center in North America. Six subjects were randomly assigned to BCG or placebo and compared to self, healthy paired controls (n = 6) or reference subjects with (n = 57) or without (n = 16) type 1 diabetes, depending upon the outcome measure. We monitored weekly blood samples for 20 weeks for insulin-autoreactive T cells, regulatory T cells (Tregs), glutamic acid decarboxylase (GAD) and other autoantibodies, and C-peptide, a marker of insulin secretion. BCG-treated patients and one placebo-treated patient who, after enrollment, unexpectedly developed acute Epstein-Barr virus infection, a known TNF inducer, exclusively showed increases in dead insulin-autoreactive T cells and induction of Tregs. C-peptide levels (pmol/L) significantly rose transiently in two BCG-treated subjects (means: 3.49 pmol/L [95% CI 2.95–3.8], 2.57 [95% CI 1.65–3.49]) and the EBV-infected subject (3.16 [95% CI 2.54–3.69]) vs.1.65 [95% CI 1.55–3.2] in reference diabetic subjects. BCG-treated subjects each had more than 50% of their C-peptide values above the 95^th^ percentile of the reference subjects. The EBV-infected subject had 18% of C-peptide values above this level.

**Conclusions/Significance:**

We conclude that BCG treatment or EBV infection transiently modified the autoimmunity that underlies type 1 diabetes by stimulating the host innate immune response. This suggests that BCG or other stimulators of host innate immunity may have value in the treatment of long-term diabetes.

**Trial Registration:**

ClinicalTrials.gov NCT00607230

## Introduction

A long-standing goal of immunology is to develop targeted immune therapies that eliminate the predominant cause of type 1 diabetes: the autoimmune T lymphocytes (T cells) that destroy the insulin-secreting cells of the pancreas. Current immune treatments for type 1 diabetes, such as immunosuppressants and anti-cytokines, are non-specific, killing or harming both the pathological T cells (i.e., insulin-autoreactive cytotoxic T cells) and healthy cells.

Two decades of autoimmune disease research in animal models, including the non-obese diabetic (NOD) mouse model of type 1 diabetes, have uncovered overlapping genetic and functional mechanisms of disease and led to the identification of the cytokine tumor necrosis factor (TNF) as a potential novel immunotherapy [1]–[7]. In the case of type 1 diabetes, the rationale for administering TNF is that insulin-autoreactive T cells bear several intracellular signaling defects that make them selectively vulnerable to death upon exposure to TNF [4]–[7]. TNF destroys insulin-autoreactive T cells, but not healthy T cells, in *in vitro* studies of human diabetic blood samples and in the NOD mouse model. TNF exposure may also augment production of beneficial regulatory T cells (Tregs), a subset of T cells believed to suppress insulin-autoreactive T cells. Interventions that have destroyed insulin-autoreactive T cells and boosted beneficial types of T cells have led to regeneration of insulin-producing islet cells in the pancreas of rodents with autoimmune diabetes, resulting in restoration of normoglycemia, even in advanced disease [7], [8].

TNF treatment at high doses in humans is limited by its systemic toxicity. An alternative approach is to test a safe, U.S. Food and Drug Administration (FDA)-approved vaccine containing *Mycobacterium bovis bacillus- Calmette-Guerin* (BCG), which has been known for over 20 years to induce TNF [9]. This avirulent strain of *Mycobacterium* is different from that which causes tuberculosis in humans (*Mycobacterium tuberculosis*).

The release of TNF after exposure to pathogens, such as BCG, is an example of a first-line host defense commonly called the innate immune response [9]. Similar results to those with TNF administration have been achieved with BCG or its non-FDA approved variant, complete Freund’s adjuvant (CFA), in rodent models of autoimmune diabetes [7], [8], [10]–[12].

Nearly two decades ago, a single, low dose of BCG in humans with late-stage pre-diabetes was initially found to successfully induce a clinical remission in some patients [13], but when efficacy was re-evaluated in expanded trials, it could not be observed a year after vaccination. At the time, the mechanisms behind BCG’s failure were not understood and specific biomarkers or knowledge of TNF action and autoimmunity were unavailable. In recent years, however, the mechanism of action underlying the therapeutic potential of BCG and TNF in autoimmune disease has been further elucidated [1], supporting the hypothesis based on animal data that BCG vaccination may be beneficial in type 1 diabetes, especially if the mechanism of action of BCG trigger TNF can be closely followed with sophisticated and early biomarkers of safety.

We conducted a proof-of-principle, double-blind, placebo-controlled trial, in which we administered two low-dose BCG vaccinations to patients with long-term type 1 diabetes. Here, we report on the safety of two low-dose BCG vaccinations and their effects on four serially studied biomarkers in long-term type 1 diabetes.

Frequent blood sampling for up to 5 months was conducted to measure biomarkers of immune and pancreatic function, including: (1) levels and viability of cytotoxic autoreactive T cells against insulin, a known autoantigen in diabetes; (2) induction of protective Tregs; (3) antibodies against the autoantigen glutamic acid decarboxylase (GAD); and (4) levels of fasting C-peptide, a marker of endogenous insulin production.

## Methods

The protocol for this trial and supporting CONSORT checklist are available as supporting information: see Checklist S1 and Protocol S1.

### Clinical Trial Participants

All clinical trial participants were required to be adults, ages 18 to 50 years, with long-term diabetes treated continuously with insulin from the time of diagnosis; have no demonstrable insulin secretion (fasting and glucagon-stimulated C-peptide less than 0.2 pmol/L) as assessed by a standard C-peptide assay by an outside vendor; be pancreatic GAD autoantibody positive; have a normal complete blood count (CBC); and have a negative purified protein derivative (PPD) test. Diabetic patients were excluded if they were pregnant or not using acceptable birth control; had a chronic infectious disease, including human immunodeficiency virus (HIV); had a history of tuberculosis (TB) or current TB infection; were currently receiving treatment with glucocorticoids, chronic immunosuppressive medications or high dose aspirin (>160 mg/day); or were currently living with an immunosuppressed individual. Also excluded were type 1 diabetics with keloid formation or hemoglobin A1C (HbA1C) values greater than 8%.

### Non-diabetic Matched Controls

Healthy, non-diabetic control subjects were included if they were 18 to 45 years of age, with no history of autoimmune disease or diabetes, no history of HIV, and no history of autoimmunity in first-degree family members. These participants were paired weekly/bi-weekly to the diabetic patients who were randomized to BCG or placebo.

### Reference Groups and Subjects

**Figure 1:**
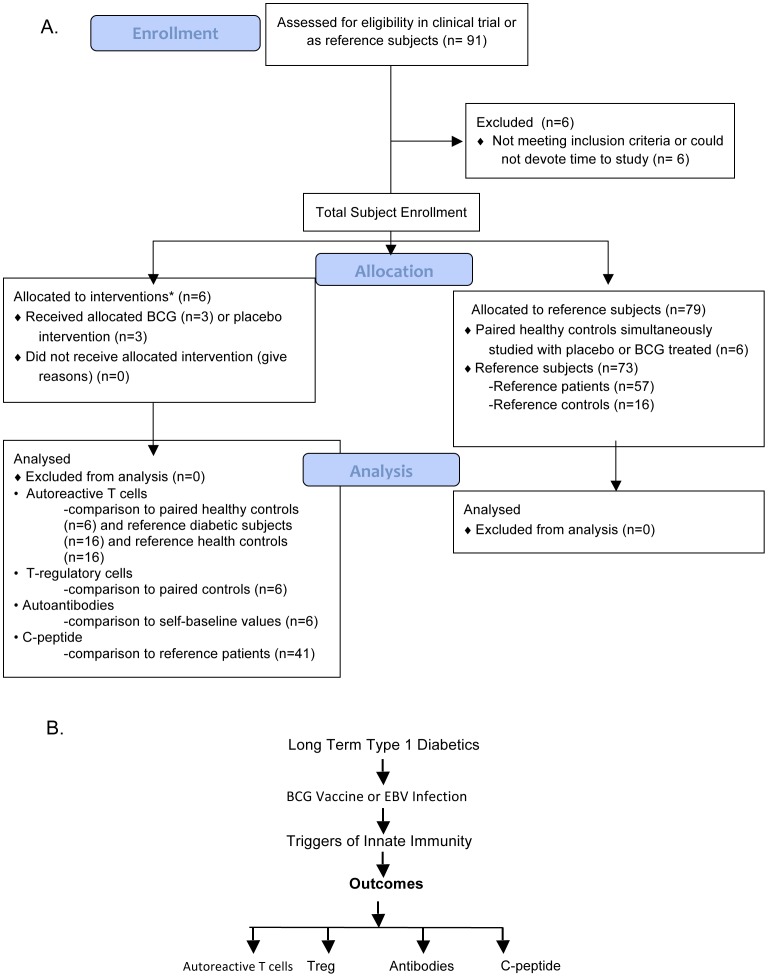
CONSORT flow chart (A) and flow diagraph (B) with depicts of treatment concept, outcomes and subject comparison groups for the study.

The study also included several reference groups: a reference group of type 1 diabetic individuals serially monitored for at least 20 weeks (n = 57) and a one-time serial studied reference group of type 1 diabetics (n = 17) studied for one outcome measure (insulin-autoreactive T cells) and matched in disease duration and age to the diabetic clinical trial subjects. The clinical trial subjects were compared to one or more of these groups, depending on the outcome measure as shown in Figure 1. The criteria for inclusion and exclusion of diabetic reference subjects were the same as those for the clinical trial subjects as related to age of onset, duration of diabetes and HbA1C values. The reference subjects studied for insulin-autoreactive T cells were also matched for human leukocyte antigen (HLA)-A2 status. The serial study of these reference subjects was performed to expand the database of autoreactive T cell variation and serially studied C-peptide values in single subjects, i.e., these separate and sequential blood draws defined the biological variation in assays in single cohorts and distinguished this biological variation from variation possibly attributable to BCG treatment in the randomized clinical trial subjects also studied in a serial fashion.

### Ethics

This study was approved by the Human Studies Committee at Massachusetts General Hospital, Boston, MA and by the FDA. All patients provided written informed consent.

### Trial Design

This was a proof-of-principle, double-blinded, placebo-controlled clinical trial that also included a paired healthy control population and reference subjects. All interventions were administered and clinical trial participants seen at one clinical center in North America (Massachusetts General Hospital, Boston, MA, USA) between 2009 and 2011. The FDA approved this protocol in 2007 and when funding was secured, the enrollment was launched in 2009.

### Intervention Population and Paired Healthy Controls

For the double-blind, placebo-controlled portion of the study, diabetic subjects were randomly assigned to BCG or placebo (saline) vaccinations according to the randomization scheme prepared by the Massachusetts General Hospital (MGH) research pharmacy. The BCG injection was prepared by the research pharmacy from lyophilized BCG (TheraCys®, Sanofi-Pasteur, Toronto, Ontario, Canada), and all syringes (BCG and saline) were prefilled by the pharmacy. Randomized patients received two 0.1 ml intradermal injections into the deltoid area containing either low-dose BCG (1.6–3.2×10^6^ colony-forming units/injection) or saline placebo, administered four weeks apart (Week 0 and Week 4). Weekly blood sampling was performed until Week 8, followed by bi-weekly blood sampling until Week 12 and then a final visit at Week 20. This frequent blood monitoring was performed to validate outcomes and observe any early effects of therapy. All subjects were seen in the morning and were required to be fasting and normoglycemic prior to having their blood drawn.

All injections were administered in the MGH diabetes clinic. Staff who administered BCG or placebo injections were not the same as those who examined the participants to grade any reactions at the injection site. All blood was processed within two hours of being drawn. All blood samples were blinded and simultaneously sent to the laboratory for monitoring of T cell response and for storage of serum for pancreas response tests (ultrasensitive C-peptide assay and autoantibodies), which were performed by outside vendors at the completion of the trial as described in “Assay methods”.

A group of paired healthy control participants, receiving neither BCG nor placebo, had blood samples obtained at the same time as diabetic subjects. Their samples were analyzed immediately for T cells in a masked fashion on the same day as the samples from diabetic subjects.

### Masking and Unblinding

The MGH research pharmacy performed all masking of BCG and saline vaccinations. All blood samples that were collected were randomly coded prior to blinded submission to the MGH lab or outside vendor lab for processing. Unblinding did not occur until all samples were processed and all data were downloaded into the central computers.

### Primary Outcome Measures

We monitored the safety of BCG in advanced type 1 diabetes and its action on immune and pancreas outcomes, including levels of insulin-autoreactive T cells, Treg cells, autoantibodies (including GAD), and C-peptide, an indicator of endogenous insulin secretion.

### T Cell Assay Methods

The two cell-based assays (Treg cells and autoreactive T cells) were performed through Week 12.

#### Cell isolation

CD4 and CD8 T cells were isolated from fresh human blood within 2 hours of venipuncture using Invitrogen™ Dynal® CD4 positive isolation kit and Dynal® CD8 positive isolation kit (Life Technologies Corporation, Carlsbad, CA, USA). This method is unique in yielding cells both free of magnetic particles and free of an attached positive selection antibody to either the CD4 protein or the CD8 protein. The blood was drawn into BD Vacutainer® tubes (BD, Franklin Lakes, NJ, USA) containing acid citrate and dextrose or ethylenediaminetetraacetic acid (EDTA). The CD8 or CD4 cells extracted for these studies were selected from fresh blood and were required, for standardization purposes, to be greater than 98% pure, 95% viable, and 85% yield for the validated T cell assays [4], [14] described below.

#### Use of controls

For all T cell assays in this study, a diabetic blood sample was always drawn at the same time as blood from a paired healthy control to allow assay standardization.

#### Detection of autoreactive CD8 T cells to a fragment of insulin

Insulin-autoreactive T cells were assayed by flow cytometry after fresh blood cell separations [14] to obtain high-yield and highly pure and viable CD8 T cells for tetramer staining. Tetramers are T cell detection reagents composed of the binding region of specific HLA class I proteins with loaded peptides in the exterior binding grooves. The tetramers, which are made fluorescent, bind to specific T cells with specific reactivity to the presented peptide fragment, thereby allowing for cell identification. To detect autoreactive T cells to insulin, we used tetramers to HLA-A2 *0210 insulin beta 10–18 with a fragment of HLVEALYLV (Beckman Coulter #T02001) [15]. To further confirm the specificity of insulin-autoreactive T cell detection, cell samples were examined simultaneously with T cell reagents to detect oncogene-specific human epidermal growth factor receptor-2 (HER-2) or Epstein-Barr virus (EBV)-specific T cells of acute infection. For simultaneously studied healthy controls, the following tetramer reagents were used: HLA*0201 Her-2/neu with a sequence to KIFGSLAFL (Beckman Coulter #T02001), a breast cancer peptide; HLA*0201 null without a non-specific peptide fragment (Beckman Coulter #T01044); or an EBV tetramer reagent HLA-A*0201 EBV with sequence of GLCTLVAML (Beckman Coulter #T01010).

Tetramer reagent staining was conducted on the highly pure CD8 T cells after 12 hours of culture at 26°C followed by 6 hours at 37°C and/or 1 hour rest at 26°C followed by 12 hours at 37°C. Cells were then stained with phycoerythrin-labeled class I tetramers (Beckman Coulter, Fullerton, CA) and SYTOX green dye (MBL International, Woburn, MA) and/or CD8 antibodies (BD Biosciences, San Jose, CA). All CD8 T cells were stained at 4°C in the dark for 30 minutes and then washed twice in Hanks balanced salt solution with 2% heat inactivated bovine serum. On average, 100,000 highly pure CD8 T cells were analyzed to ensure optimized data points on the Becton Dickinson FACSCalibur using the Cell Quest acquisition program and allow the detection of rare autoreactive T cells. All cells were analyzed while fresh to prevent fixation artifacts and enable quantification of dead versus viable cells. Prior to tetramer staining, cells were neither frozen nor expanded. Calculations of insulin-positive T cells were reported as the percentage of insulin-autoreactive T cells to the total numbers of isolated pure CD8 T lymphocytes.

Note that all diabetic treated patients in the randomized portion of the study were HLA-A2+ except for diabetic #iv. Although diabetic #iv was HLA-A2 negative, the formal binding site for the HLA-A2 insulin-autoreactive T cell reagent was HLA-A6802. HLA-A6802 is a subtype of the HLA-A2 family and has an identical binding cleft to HLA-A2 and other common subtypes within the HLA-A2 family. Therefore, if diabetic subject #iv were to have detectable insulin-autoreactive T cells, those cells would stain positive for the insulin-autoreactive T cell reagent. Three healthy controls in this study (Control #ii, Control #iii and Control #v) were also HLA-A2+.

Reference diabetics were monitored over a three-year period for the presence or absence of insulin-autoreactive T cells and compared to their paired healthy reference controls.

#### Detection of Treg CD4+ cells

Treg cells were assayed by flow cytometry after fresh blood cell separations as described above and by Burger et al [14]. Two different methods of cell detection were employed. Treg cells were detected as either CD4, CD25^bright^ with Foxp3 staining, or with CD4, CD25^bright^ and CD127^low^antibody staining. Intracellular staining of Foxp3 was performed with Human Treg Flow™ Kit (Biolegend, San Diego, CA, USA), according to the manufacturer’s instructions. Isolated CD4 positive cells were incubated briefly with CD4-PE-Cy5 (clone RPA-T4) and CD25-PE (clone BC96) antibodies for 20 minutes at room temperature. After washing, cells were fixed with Foxp3 Fix/Perm solution (Biolegend) for 20 minutes at room temperature. Cells were washed again and permeabilized with Foxp3 Perm Buffer (Biolegend) for 15 minutes at room temperature. Cells were then stained with Foxp3 Alexa Fluor® 488 antibody (clone 259D, Biolegend) for 30 minutes. Isotype controls were done for each sample prior to flow cytometric analysis. For detection of Treg cells, staining was performed with a CD4 antibody (clone RPA-T4, BD Biosciences, San Jose, CA, USA), a CD25 antibody (clone 4E3, Miltenyi Biotech, Auburn, CA, USA) and an anti-human CD127 antibody (clone hIL-7R-M21, BD Biosciences).

#### Flow cytometry for T cell assays

For the flow cytometry studies, the flow gates were set “open” for inclusion of CD8 or CD4 T cells of all sizes, but exclusion of the following: cell debris, red blood cells, fragmented cells, and apoptotic bodies. The “open gate” was chosen for the purified CD8 or CD4 T cells because T cells undergoing cell death, especially by apoptosis, can display changes in light scattering properties. The goal was to ensure accuracy by analyzing high numbers of cells per sample and to capture dying cells of all shapes. Cell viability was quantified by either of two stains that fluorescently labeled dead cells, i.e., Sytox (MBL international Co., Woburn, MA, USA) or propidium iodine (PI). Purified CD8 cells form distinct scatter pictures on forward versus side scatter highlighted the shrunken size of dead versus viable cells.

With open gating and inclusion of all purified CD8 T cells in each sample, some reference diabetics consistently displayed insulin-autoreactive T cells. In contrast, some reference diabetics consistently had undetectable insulin-autoreactive T cells compared to healthy reference controls, which were simultaneously studied at each monitoring time. The data were collected over the multi-year time span. The signal for insulin-autoreactive T cells was in the range of 0.06–0.09%. The healthy control background signal is in the range of 0.04–0.05% [15]. The reverse was also true: diabetics who initially lacked insulin-autoreactive T cells, on repeat sampling, continued to lack those cells.

### Serum Assay Methods for Pancreas Monitoring

GAD autoantibody and fasting C-peptide levels were assayed by radio-binding and ELISA assays in diabetic subjects to assess whether the subjects had a pancreas response to the BCG injection. For these serum assays, fresh human blood was collected by venipuncture into red top tubes and allowed to clot. The serum was then separated by centrifugation within 2 hours of venipuncture. Serum was stored at −80°C until analysis. The C-peptide assay was undertaken through week 20.

#### Detection of C-peptide secretion

Measurement of connecting peptide (C-peptide) co-secreted with insulin permits direct estimation of any remaining insulin from the pancreas in contrast with endogenous sources. The first, performed by the Mayo Clinic (Rochester, MN, USA) utilizing the Roche Cobas C-peptide assay (Roche Diagnostics, Indianapolis, IN, USA) for clinically detectable C-peptide, was used for eligibility and had a lower limit of detection of 330–470 pmol/L. This insensitive but standard assay was applied to fasting and glucagon-stimulated blood samples. After screening negative for enrollment purposes, subjects’ serum was stored and freezer-banked. For subsequent samples (baseline through Week 20), the saved serum was sent to Sweden for analysis of serial C-peptide levels by an ultrasensitive C-peptide assay with a lower level of detection of 1.5 pmol/L and an assay range up to 285 pmol/L (Mercodia AB, Uppsala, Sweden). For C-peptide values of 1.5–37 pmol/L, the within-assay coefficient of variation was 3.8%; for values of 38–60 pmol/L, it was 2.6%; and for values of 143–285 pmol/L, it was 2.5%. The Mercodia Ultrasensitive C-peptide ELISA kit, which is an FDA-listed reagent and has a filed document registration, has been evaluated for accuracy and is classified in the United States as a class one device for ultrasensitive detection of C-peptide levels. This assay is calibrated against the International Reference Reagent for C-peptide, IRR C-peptide 84/510. All statistics on C-peptide levels were performed using the lower level of detection of the assay, i.e., 1.5 pmol/L.

#### Detection of GAD autoantibodies

GAD autoantibodies provide evidence of diabetic autoimmunity since GAD proteins are intracellular proteins specific to insulin secreting cells and are released from T cell mediated beta cell destruction. The release of intracellular GAD results in the immune response of autoantibodies. Enrolled patients were required to be GAD autoantibody positive. Prior to enrollment, a single serum sample for GAD autoantibody was sent either to the Joslin Clinic in Boston, MA, USA (Subject #vi, Subject #i, Subject #ii, Subject #iv) or to Quest Diagnostics (Cambridge, MA, USA) (Subject #iii, Subject #iv). After the first BCG or placebo injection, serum samples collected from baseline to Week 20 were sent to Germany for diabetic autoantibody panels [16] at the laboratories of Drs. Ezio Bonifacio and Peter Achenbach of the Diabetes Research Institute in Munich, Germany. The autoantibodies studied were GAD, IA-2A (islet-specific protein tyrosine phosphatase), and ZnT8Carg-A (pancreatic beta cell-specific zinc transporter) [17]. The GAD assay sensitivity is 86%, specificity is 100%, and inter-assay variation is 18%. For the IA-2 autoantibody assay, the sensitivity is 72%, the specificity is 100%, and the inter-assay variation is 16%. For the ZnT8Carg-A assay, the sensitivity is 72%, the specificity is 99%, and the inter-assay variation is 17%.

### Sample Size

Sample size for the randomized population was determined in conjunction with the FDA and with the intense use of serial biomarker studies as outlined by the Institute of Medicine guidelines for clinical trials [18]. A sample size of 6 randomized patients was determined as appropriate for the intense serial blood monitoring required in this proof-of-concept trial for the placebo or BCG interventional limbs and an expanded population of diabetics and non-diabetic controls for assay validation that is referred to as reference subjects.

### Statistical Analysis

Randomized participants were compared to self, healthy paired controls, or reference subjects with or without type 1 diabetes, depending on the outcome measure, according to the schema depicted in Figure 1. None of the analyses compared the BCG-treated to placebo-treated clinical trial subjects.

For each randomized patient, a linear regression model with auto-correlated errors was used for statistical comparisons between baseline and post-treatment periods in autoantibody levels over the course of the study. This was the appropriate test for this comparison because any change in autoantibodies should be sustained over the monitoring period of this trial, i.e., the t _1/2_ of B cells that produce antibodies exceeds 60 days. P-values compared the values of each person to their post-baseline values by two-sided test based on a regression model with auto-correlated errors. For C-peptide assays, a cut-off value of 1.5 pmol/L was used since this value is the lower limit of detection of the ultra-sensitive assay used in this study. C-peptide assays were performed by the outside vendors in duplicate; figures are therefore presented as the means +/− the SE. For the comparison of EBV-infected or BCG-injected patients to the long-term diabetic reference samples, the Kolmogorov-Smirnov two-sample test was used to compare the distribution of each patient with the reference samples. We applied this method in a conservative fashion by overestimating the variability of the clinical trial sample, as a more exact comparison is difficult to obtain due to the low sampling frequency and small numbers of measurements per patient in the reference group. P-values of <0.05 were considered statistically significant. SAS® version 9.2 was used for the statistical analysis.

For serum samples sent out to commercial sources for assay performance, both published inter-assay and intra-assay variability was considered for the statistical analysis of the clinical trial samples. We also verified that the inter-assay variability was consistent in the plate for the clinical samples by comparing the pre-treatment values with all post-treatment values of the same patient to self in the same plate. This self-comparison analysis was performed for serum assays such as C-peptide or autoantibodies. The area under the curve (AUC) was calculated for all treatment and control groups, although the control group varied according to the assay.

## Results

### Participant Enrollment and Characteristics

**Figure 2:**
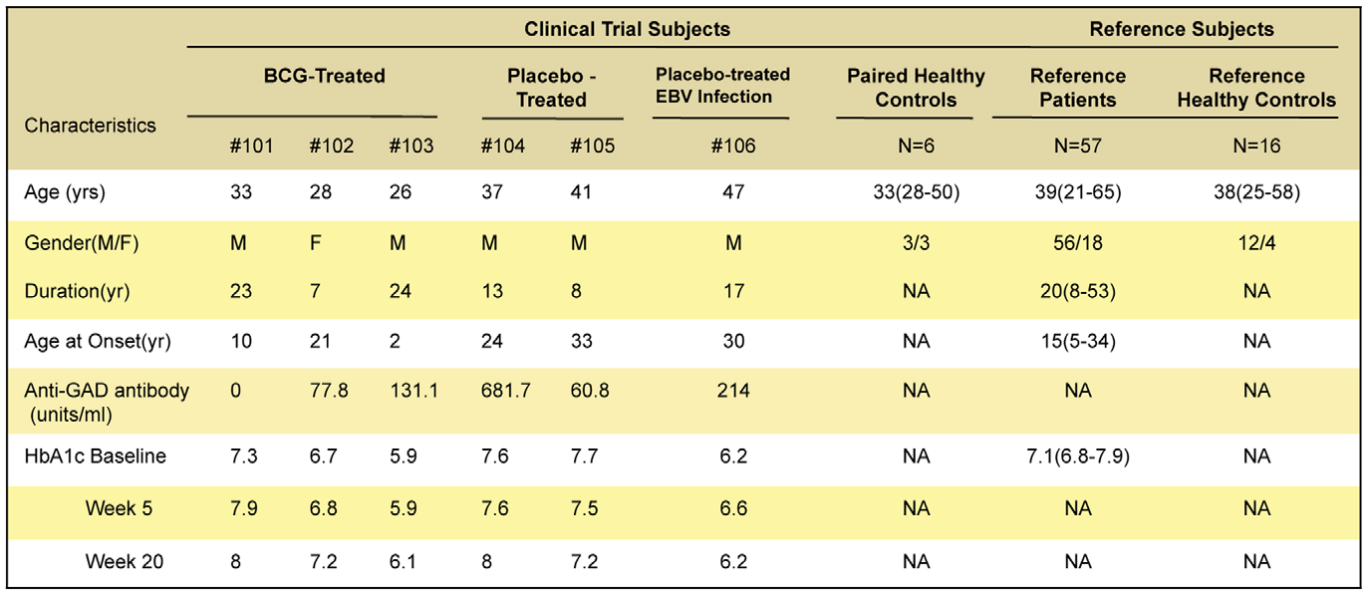
Clinical characteristics of groups of clinical trial subjects and reference subjects.

A total of 85 participants were studied: 63 type 1 diabetics and 22 non-diabetic controls (Fig. 1, Fig. 2.). In the double-blinded, placebo-controlled portion of the study, a total of six diabetic subjects were randomly assigned to BCG or placebo vaccinations. The randomized clinical trial subjects had disease for a mean duration of 15.3 years (range 7–23 years) and mean age of 35 years (range 26–47) (Fig. 2), and were paired to healthy controls (n = 6) at each weekly blood drawing time for greater than 20 weeks of study. In addition to these participants, 57 additional reference subjects with long-term diabetes and 16 reference healthy subjects served as reference subjects for both serial T cell assays and serum sample comparisons. Diabetic reference patients had disease for a mean duration 20 years (range 8–53 years) and mean age of 39 years (range 21–65) (Fig. 2). The intense serial monitoring of blood samples of all clinical trial subjects resulted in a total of 1,012 blood samples from diabetic or comparison subjects to quantify both T cell and pancreas changes. This serial study of biomarkers and comparison groups for the subjects are depicted in Fig. 1. This intensive study of novel T cell and pancreas biomarkers required different comparison groups (Fig. 1) due to the lack of serial normative data on the four parameters chosen to study BCG efficacy in advanced type 1 diabetes. The objective of the trial was to test safety of multi-dosing BCG in long-term diabetics. Four monitored endpoints of efficacy were studied as markers of disease activity: death of insulin autoreactive T cells, induction of Treg cells, changes in autoantibodies and the restoration of endogenous insulin secretion through C-peptide levels.

### Epstein-Barr Virus (EBV) Infection

**Figure 3:**
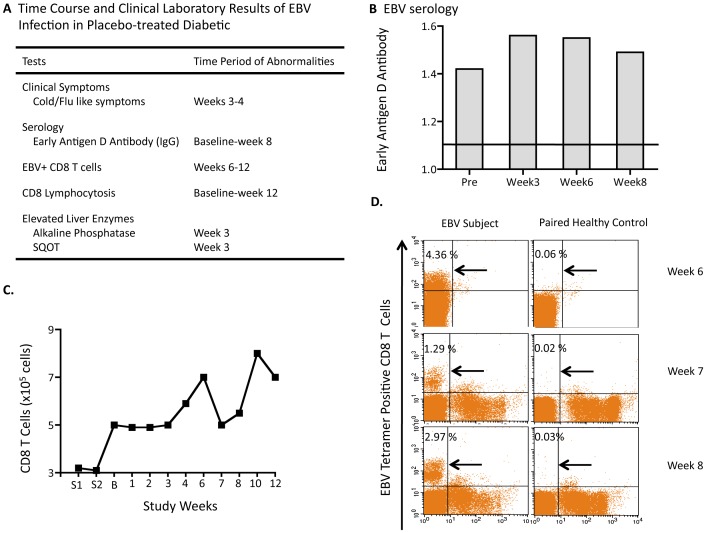
Clinical laboratory studies reveal acute EBV infection in placebo-treated diabetic. (**A**) Weekly course of EBV infection from serum of diabetic subject #vi (**B**) Positive Early Antigen D antibody versus negative values. (**C**) CD8 T-cell proliferative response. (**D**) Flow scatter plots of appearance of EBV-reactive T-cells vs. paired control, week 6 to 8. All newly appearing EBV-reactive T-cells were viable.

At screening for clinical trial enrollment, and unbeknownst to us, one diabetic clinical trial subject had an acute undiagnosed case of EBV infection. This patient presented with cold/flu symptoms at weeks 3–4 after the placebo injection (Fig. 3). The presence of the new EBV infection in blood samples was detected during our blinded laboratory protocols that required analysis of EBV-reactive T cells (EBV-tetramer positive CD8 T cells) as a control during the CD8 insulin-autoreactive T cell assays. Further confirmation of this diagnosis of acute EBV infection was obtained at the end of the trial with serology sent for commercial antibody testing (Quest Diagnostics, Cambridge, MA, USA) (Fig. 3).

This placebo-treated EBV subject completed the five-month trial and was subjected to the same types of statistical analyses and outcome studies as other clinical trial subjects. The treatment team and subject remained masked to treatment assignment.

The course of the EBV infection was reconstructed from serially studied fresh T cell samples and by standard clinical laboratory tests on stored serum samples (Fig. 3). To understand the precise time course of the EBV infection, this diabetic’s serum was screened for EBV VCA antibody (IgM), an antibody that is typically positive days after infection onset to 3–6 weeks post-infection. The serum was also tested for EBV Early Antigen D Ab, an antibody that is typically positive only in the infection window running from 1 month after infection to 2 months post-infection (Fig. 3).

This placebo-treated diabetic subject was early antigen D antibody-positive at the first baseline sample at week 0, had CD8 lymphocytosis over 12 weeks of study (Fig. 3) and demonstrated mildly elevated liver enzyme levels early in the trial course, all consistent with an acute EBV infection. As the EBV serologic studies show, Subject #vi had an acute infection that lasted longer than one month but did not exceed two months in duration. The EBV tetramer positive cells became vividly positive at week 6 in the T cell assay and were still vividly positive at week 8, although declining slightly (Fig. 3). As a comparison, we include the EBV positive data from a long-term diabetic that was not part of this clinical trial, but who had a very distant past EBV infection, to show the low numbers of EBV memory cells seen using the EBV tetramer methods when infection is not acute (Fig. S1).

All other clinical trial subjects in this study were negative for both acute and past EBV infections throughout the duration of T cell monitoring during the trial (Fig. S1). EBV infections, like BCG, trigger innate immunity by inducing secretion of host TNF [9]. The patient’s EBV status and receipt of placebo saline injections fortuitously enabled us to compare the serial T cell and pancreas effects of EBV- and BCG-triggered innate immune responses in the same study [9], [19]. All other clinical trial subjects in this study were negative for both acute and past EBV infections through T cell monitoring during the trial (Fig. S1).

### The Majority of Insulin-autoreactive T Cells Released into the Blood after BCG Treatment or EBV Infection are Dead

**Figure 4:**
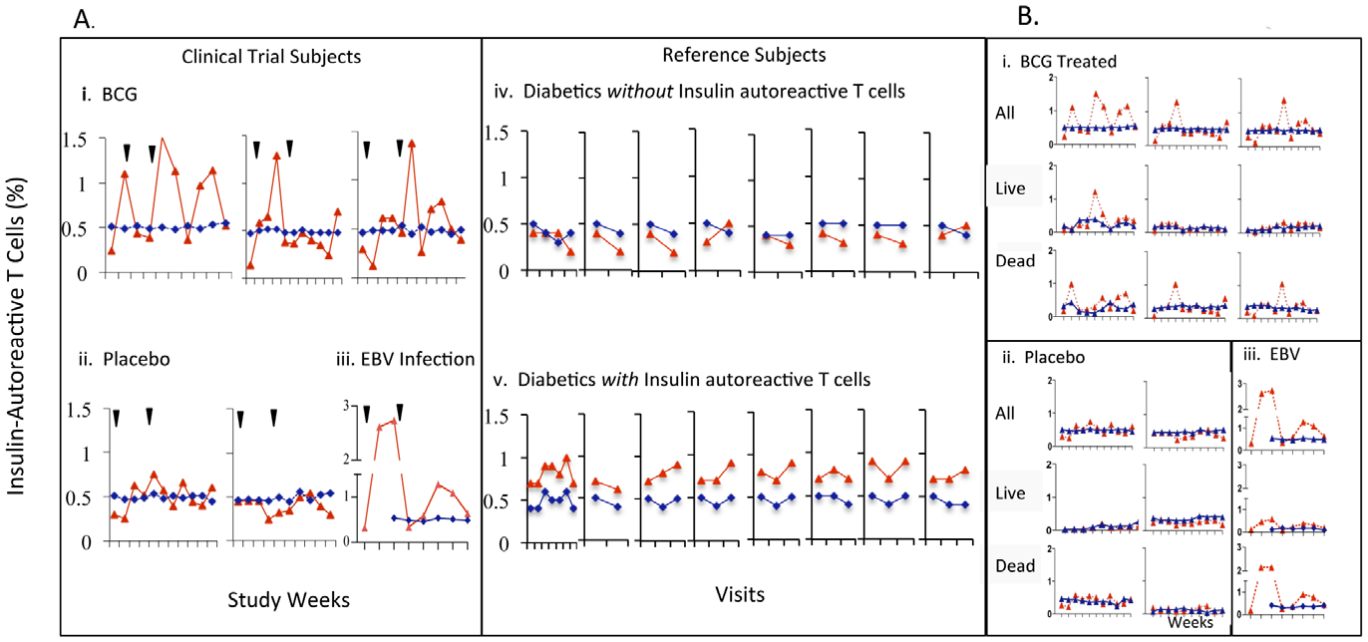
Insulin-autoreactive T-cells released into the circulation are dead after BCG treatment or EBV infection. (A) Percentage of insulin-autoreactive T-cells of total CD8 T-cells over 12 weeks for BCG-treated (***Ai, Bi***), placebo-treated, (***Aii, Bii***) and EBV-infected clinical trial subjects (***Aiii, Biii***). Reference diabetics without or with insulin-autoreactive T-cells vs. reference healthy controls (***Aiv,v***). (**B**) Insulin-autoreactive T-cells stratified by viability in clinical trial subjects. Red diamonds are long-term diabetics; blue diamonds paired healthy controls. Arrows are BCG or placebo injection times.

At baseline, all six clinical trial subjects lacked elevated levels of insulin-autoreactive T cells compared to their paired non-diabetic controls, with ≤0.4% as the upper limit of normal based on the reference subjects and background staining (Fig. 4). The presence of insulin-autoreactive T cells was not a requirement for enrollment into this study, and past studies identified pathologic autoreactive T cells reactive with this peptide in about 40% of long-term diabetics [4]. Within 1 to 4 weeks after BCG treatment, increased numbers of insulin-autoreactive T cells appeared in the circulation of each BCG-treated subject vs. their paired healthy control (Fig. 4*A* *i*). Similar, if not greater elevations in circulating insulin-autoreactive T cells were also seen in the EBV-infected placebo subject coincident with the T cell and serologic immune response to an ongoing EBV infection (Fig. 4*A* *iii*). Like the non-EBV infected placebo-treated subjects (Fig. 4Aii), all paired healthy controls showed no change (Fig. 4*A* *i–iii*, blue lines).

Among diabetic reference subjects, approximately 60% had no insulin-autoreactive T cells. Their values ranged from 0.2–0.4% at all determinations, levels essentially indistinguishable from their paired non-diabetic controls (Fig. 4*A* *iv,v*). The remaining 40% consistently had insulin-autoreactive T cell levels ranging from 0.4–1% at all measurements, a range higher than their paired non-diabetic controls (Fig. 4*A* *iv,v*). None of the diabetic reference subjects followed longitudinally and having baseline insulin-autoreactive T cells of <0.4% (n = 8) had subsequent values that rose above 0.4%. Thus, the presence or absence of circulating insulin-autoreactive T cells was shown to be a stable phenotype in serially studied and untreated type 1 diabetic subjects with these monitoring methods.

**Figure 5:**
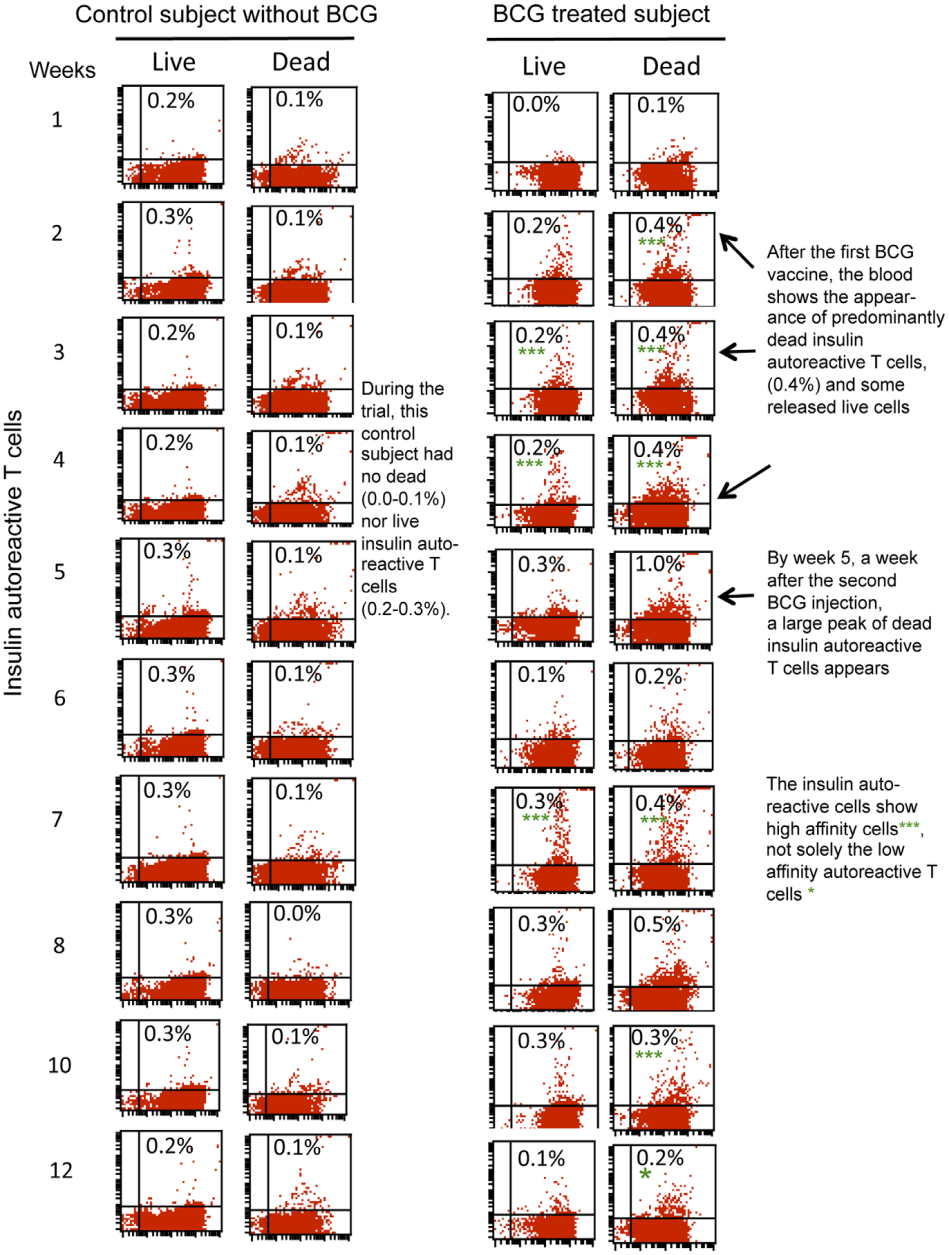
Two-color flow pictures of the serial weekly blood monitoring of dead and live insulin autoreactive T cells in a control subject (left) and BCG-treated diabetic subject (right). After the first BCG treatment, predominantly dead insulin-autoreactive T cells appear in the circulation of the diabetic compared to the simultaneously studied paired healthy control. For all recruited BCG-treated diabetic subjects, the start of the trial shows fresh blood samples with no insulin-autoreactive T-cells in these longterm diabetics, followed by dead insulin-autoreactive T-cells that persist through week 4, recurrent dead insulin-autoreactive cells released again after the second injection of BCG followed by the gradual disappearance of the dead insulin-autoreactive T-cells by week 12 of monitoring. It should be noted that the newly released insulin-autoreactive cells after BCG are unique in representing both low affinity (*) autoreactive T-cells that can be observed in the routine monitoring of positive patients and high affinity(***) autoreactive T-cells that are never observed in routine monitoring of diabetic patients. In contrast to the serial monitoring of a BCG treated subject, the serial studied fresh blood samples of the control subject reveal throughout the study the lack of either live or dead insulin-autoreactive T-cells.

The insulin-autoreactive T cells appearing in the circulation after BCG or EBV infection were more likely dead than alive compared to paired healthy controls (Fig. 4, Fig. S2, Fig. 5), probably indicating not only the rapid release of pre-formed insulin-autoreactive T cells after BCG treatment or EBV infection but also their redundant death by TNF induction. Also unlike the low affinity insulin-autoreactive T cells observed with routine monitoring of diabetics, the TNF-targeted death of pathogenic cells allowed the identification of both low affinity as well as newly appearing, high affinity subsets of autoreactive T cells not previously identified in the circulation (Fig. 5). For the three BCG-treated subjects, the AUC representing the cumulative concentrations of insulin-autoreactive T cells over the course of study were 2.22, 0.71 and 1.03 compared to their paired healthy control. The two non-EBV infected placebo-treated subjects’ AUCs were 0.57 and 0.07, while the EBV-infected subject had a strikingly elevated AUC of 5.69, reflecting the large numbers of dead insulin-autoreactive T cells being released into the circulation after the EBV infection. The transient increases in the number of insulin-autoreactive cells seen in the BCG-treated or EBV-infected clinical trial subjects (Fig. 4*A* *i, iii*) formed a pattern distinctly different than the stable levels observed in the two other placebo-treated subjects (Fig. 4*A* *ii*) and in reference diabetic subjects (Fig. 4*A* *iv,v*). Cytometric study of dead and living insulin-autoreactive T cells revealed that the pathogenic T cells captured in the blood had both the common low affinity insulin-autoreactive cells as well as the treatment-specific release of high affinity autoreactive T cells for the insulin peptide fragments (Fig. 5). Routine monitoring of diabetics for insulin autoreactive T cells by diverse studies only reveals low affinity insulin-autoreactive T cells in diabetes subjects without treatment [4]. The TNF-induced death *in vivo* of insulin-autoreactive T cells with BCG vaccinations or acute EBV infection was confined to the autoreactive T cells.

### Regulatory T Cells are Induced by BCG and EBV

**Figure 6:**
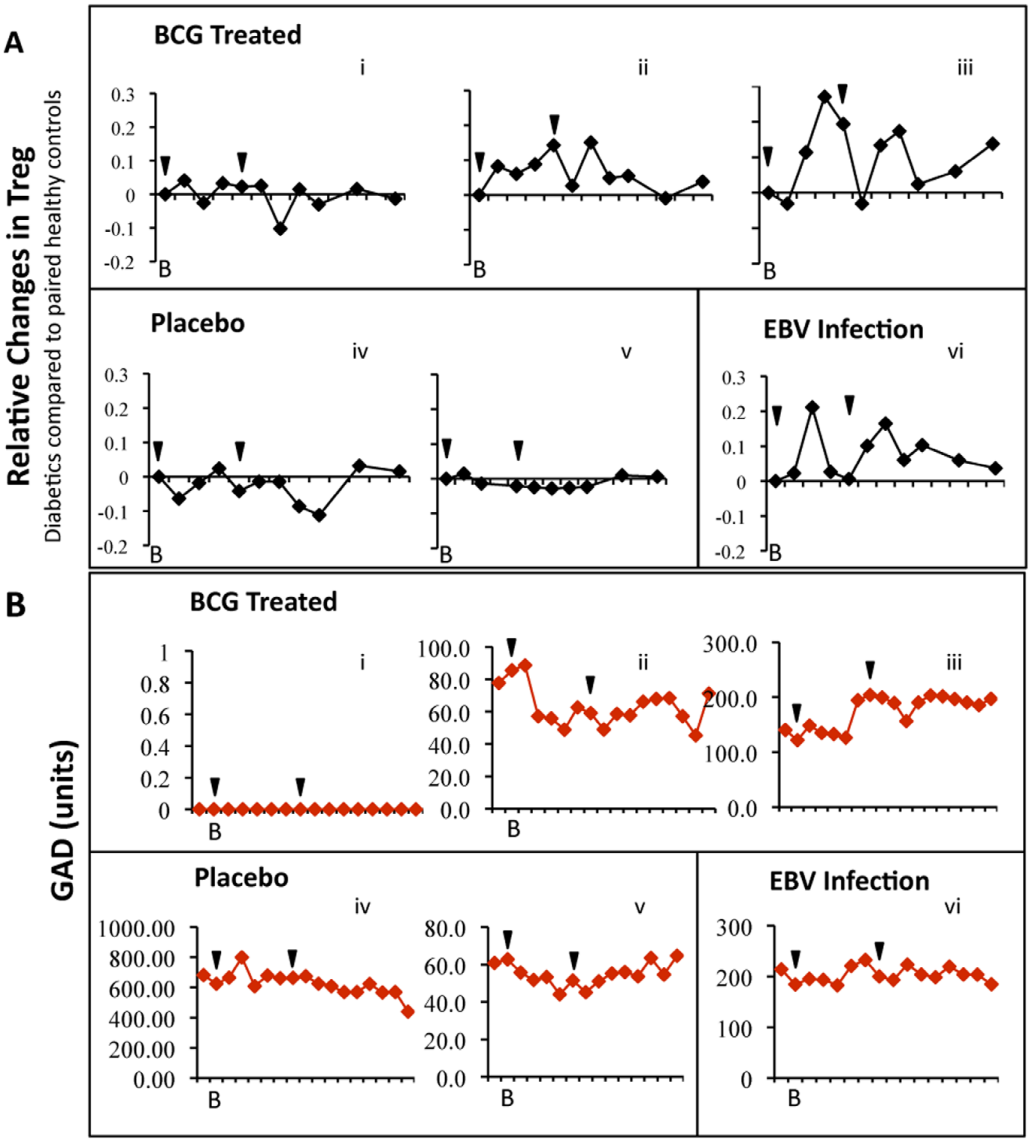
T_REG_ cells and GAD-autoantibodies change in response to BCG and EBV. (**A**) T_REG_ cell ratios in BCG-treated, placebo, and EBV-infected clinical trial subjects by week vs. paired healthy controls. (B) GAD autoantibody levels vs. own baseline in BCG-treated placebo-treated, and EBV-infected clinical trial in each subject, by week. B is baseline prior to trial. Arrows are BCG or placebo injection times.

The EBV-infected subject and two BCG-treated subjects appeared to exhibit increases in the numbers of Treg cells compared to their paired healthy controls studied simultaneously (Fig. 6*A* *ii, iii, vi*); the other two placebo-control subjects had stable levels (Fig. 6*A* *iv, v*). A similar trend for elevations in Tregs in response to BCG or EBV was observed by measuring the AUC, a measure of the total accumulation of Treg ratios. The three BCG-treated subjects had cumulative Treg ratios of patients compared to controls of 0.12, 0.42 and 0.30 compared to placebo treated subject accumulations of 0.11 and 0.03. The EBV infected subject had cumulative Tregs of 0.32.

### GAD Autoantibody Levels Show Sustained Change after BCG Treatment

**Figure 7:**
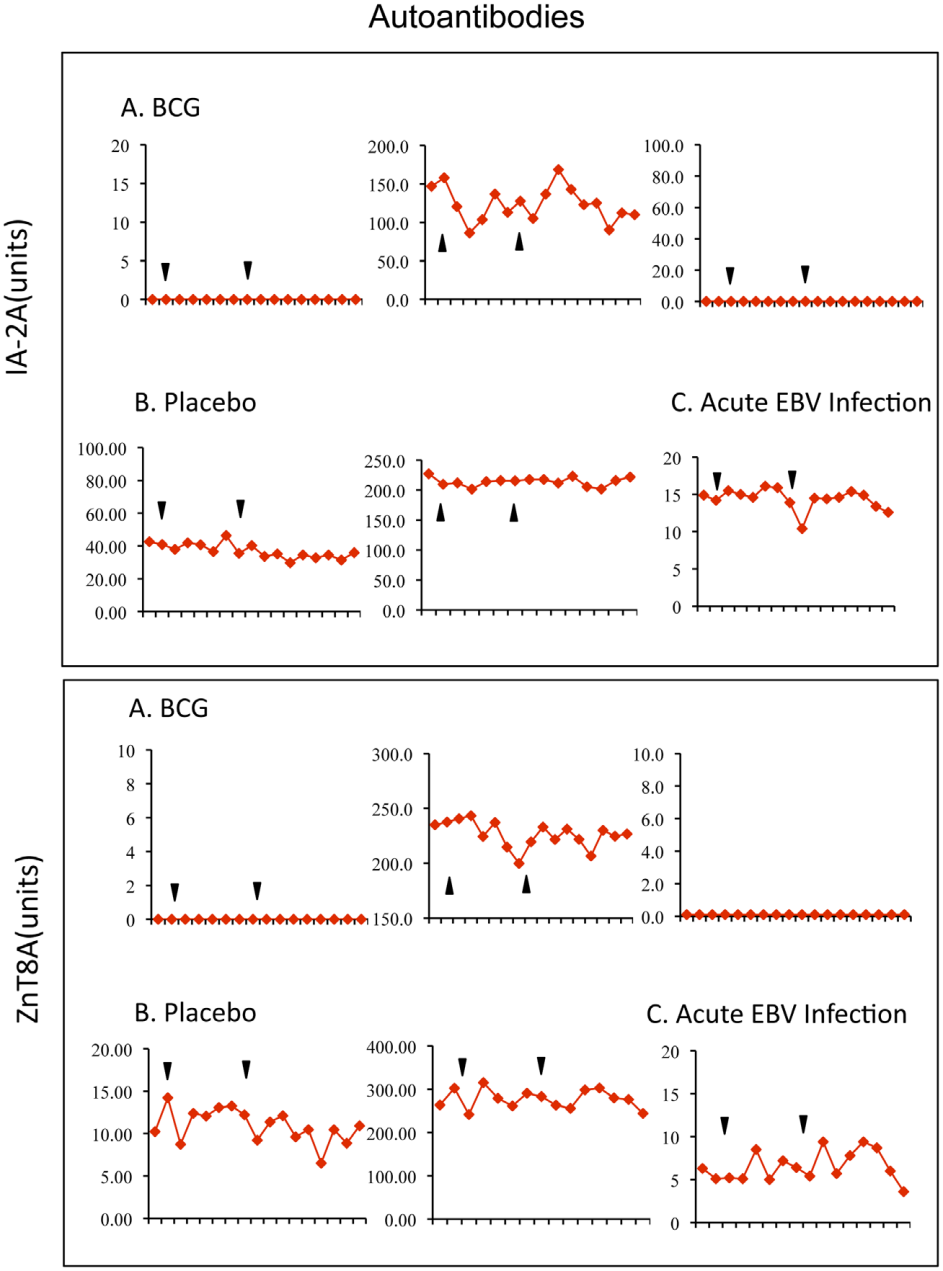
IA-2A and ZnT8 autoantibodies in clinical trial subjects by study week.

At baseline, GAD autoantibodies, ranging from 60 to 650 units, were present in all diabetic clinical trial subjects except one BCG-treated subject (Fig. 6*B*). There was a statistically significant and sustained change in GAD autoantibody levels in two of the three BCG-treated subjects after injections, with one diabetic showing a decrease and the other an increase relative to self-baseline (p = 0.0001 and p = 0.0017, respectively (Fig. 6*B* *ii,iii*). In contrast, none of the other diabetic subjects showed any variations from their baseline values of GAD (Fig. 6*B* *iv,v,vi*). The other islet-specific autoantibodies studied, tyrosine phosphatase IA-2A and beta cell-specific zinc transporter (ZnT8A), were present in some of the diabetic subjects at baseline (Fig. 7); only ZnT8A had statistically significant decreases in one BCG treatment subject. A similar trend for higher or lower acute elevations in GAD in response to BCG was observed by measuring the AUC, a measure of the total positive or negative accumulations of GAD autoantibody levels over the course of the trial. The total raw levels of GAD autoantibodies over the trial course were 0.00, −379 and +433 for the BCG-treated subjects and −102 and −116 for the placebo treated subject. The EBV-subject accumulated GAD autoantibodies of 245. Altered GAD autoantibody levels have been documented to decrease after re-exposure of the immune system to childhood BCG vaccinations and acutely increase or decrease after islet transplantation although the clinical significance is unknown [20]–[22].

### Fasting Insulin Secretion Temporarily Increased as Measured by C-peptide after BCG and EBV Infection

**Figure 8:**
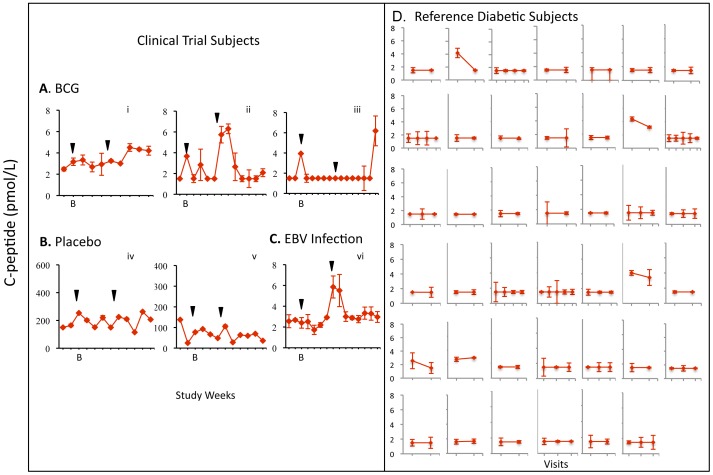
Fasting C-peptide levels show transient increase in BCG-treated and EBV-infected clinical trial subjects. Fasting C-peptide for (**A**) BCG-treated, (**B**) Placebo-treated, and (**C**) EBV-infected clinical trial subjects by week vs. (**D**) Reference diabetics, by visit. C-peptide levels are measured by ultrasensitive C-peptide assay in duplicate. Arrows are BCG or placebo injection times.

At baseline as a recruitment requirement, none of the six diabetic clinical trial subjects had detectable levels of fasting or stimulated C-peptide using a relatively low sensitivity C-peptide assay for screening in the standard clinic setting. Serum from all clinical trial patients was saved for subsequent insulin secretion studies with an ultrasensitive C-peptide assay. When the baseline samples were re-assayed with the ultrasensitive assay, all six clinical trial subjects had detectable C-peptide above the lower range of sensitivity of the ultrasensitive assay (>1.5 pmol/L) (Fig. 8).

**Figure 9:**
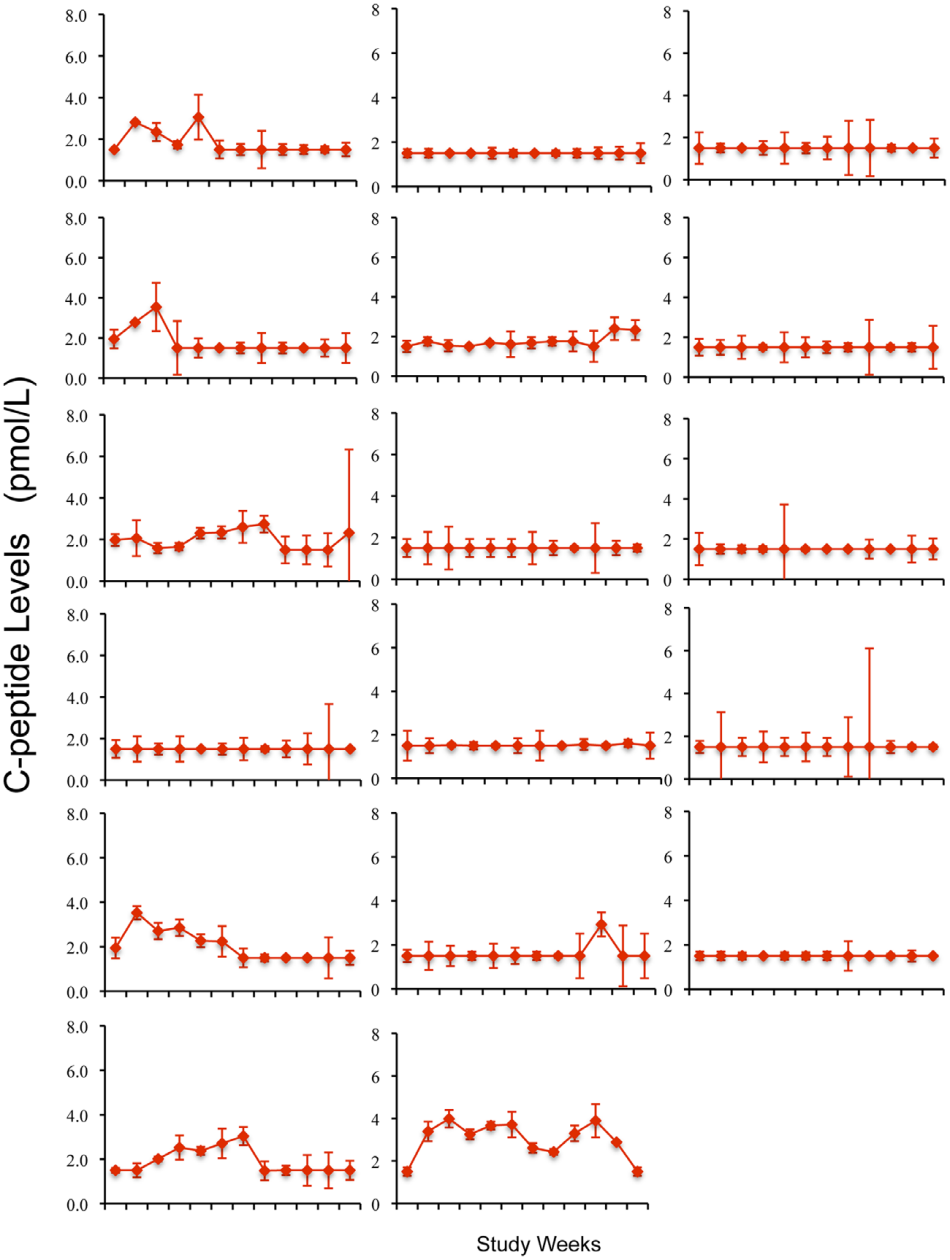
C-peptide levels remain stable and near the lower limit of an ultrasensitive assay in a longterm diabetic group (N = 17) sampled weekly for 12 weeks in a fasting state.

Two of the three BCG-treated subjects and the EBV-infected subject had transient increases in fasting C-peptide levels by Week 20 compared to either their baseline or to the values in 41 reference diabetic subjects. Specifically, C-peptide levels transiently and significantly rose with BCG administration in Subject #i (mean concentration 3.49 pmol/L [95% CI 2.95–3.8]), Subject #ii (2.57 pmol/L [95% CI 1.65–3.49]), as well as in the EBV-infected placebo Subject #vi (3.16 pmol/L [95% CI 2.54–3.69]) relative to 41 reference diabetic subjects (mean = 1.65 pmol/L [95% CI 1.55–3.2]), using the Kolmogorov-Smirnov two-sample test (Fig. 8). Subjects #i and #ii each had more than 50% of their C-peptide values above the 95^th^ percentile of the reference levels. Subject #vi had 18% of C-peptide values above this level. Neither non-EBV infected placebo-treated diabetic subject (*iv and v*) had C-peptide fluctuations of statistical significance**.** The biologic stability of low levels of fasting C-peptide levels with serial monitoring in the ultra-sensitive assay is apparent in 41 reference diabetics (Fig. 8*D*) and confirmed in 17 additional diabetic subjects evaluated weekly for 12 weeks that were collected after the trial completion to further confirm the stability of the ultrasensitive C-peptide assay in serially studied long term diabetics with these low levels (Fig. 9). AUC measurements of C-peptide, a measure of cumulative changes of C-peptide levels over the 5-month trial, were higher in the two BCG-treated and one EBV-infected subject than in the non-EBV infected placebo clinical trial subjects.

### Other Clinical and Safety Monitoring

There were no significant changes in any of the clinical trial patients in any safety monitoring parameters, including routine chemistry and liver function tests, hematologic studies, or HbA1c levels. Other than the expected vaccination scars associated with BCG, no adverse effects occurred. None of the participants dropped out of the clinical trial.

## Discussion

The goals of the current trial were to determine whether activation of the innate immune system could be accomplished safely with repeated BCG vaccinations and whether this treatment would ameliorate, for any time period, the advanced autoimmune state of long-term type 1 diabetes. We found that repeated BCG vaccination at low doses was safe and well tolerated. We also found that BCG vaccination and an unexpected EBV infection in a placebo-treated diabetic subject, both known triggers of innate immunity, caused rapid increases in circulating insulin-autoreactive T cells that were mostly dead. The rapid release of dead insulin-autoreactive T cells supports the hypothesis, first demonstrated in the NOD-mouse model of autoimmune diabetes, that BCG ameliorates the advanced autoimmune process underlying type 1 diabetes by stimulating TNF, which selectively kills only disease-causing cells and, further, permits pancreas regeneration [7], [8] as evidenced by the transient increase in C-peptide secretion we observed using an ultrasensitive C-peptide assay.

The response we observed in the placebo subject who experienced an acute EBV infection provides evidence that infectious agents other than *Mycobacterium* can activate innate immunity in long-term diabetic subjects and modify the host’s aberrant autoimmune response [9]. The subjects EBV status and receipt of placebo saline injections fortuitously enabled us to compare the serial T cell and pancreas effects of EBV- and BCG-triggered innate immune responses in the same study [9], [19]. EBV infections, like BCG, are known to trigger innate immunity by inducing a strong host TNF response [9], [19], and the changes in autoimmune cells and beta cell responses we observed in BCG-treated subjects were similar or sometimes even larger in the EBV-infected subject, suggesting that a larger dose of BCG might be more effective. The transient increases in C-peptide, found after both an acute EBV infection and with BCG vaccinated subjects, suggests a positive impact of these immune perturbations on beta cell function.

This study may offer mechanistic insights into ongoing clinical trials of broad-spectrum immunosuppressive drugs, such as anti-CD3 antibodies, in new-onset type 1 diabetes. The administration of humanized anti-CD3 antibodies is associated with side effects, including re-activation of EBV in recent-onset type 1 diabetes. as reported to the FDA. Lowering the dose of anti-CD3 antibodies reduced EBV reactivation in clinical studies, but also eliminated efficacy. In another trial of anti-CD3 in new-onset diabetes, the release of greater numbers of insulin-autoreactive-specific T cells correlated with the simultaneous appearance in the circulation of EBV-specific T cells. Taken together, findings from anti-CD3 trials and the trial reported in this paper demonstrate that EBV infection or BCG vaccination marshals innate immunity characterized by known elevations in TNF and that this leads to potentially therapeutic benefits, especially death of insulin-autoreactive T cells.

Drug development is facilitated by understanding drug mechanism and by development of biomarkers for monitoring early responses to therapy. One previous uncontrolled study of a single dose BCG vaccination reported possibly successful stabilization of blood sugars in 65% of pre-diabetic patients [13]. Subsequent controlled clinical studies of a single low-dose BCG vaccination in new-onset diabetic children did not show a benefit when the patients were re-studied, typically a year later [23]–[25]. The current trial is unique in now understanding the mechanism of BCG and the development of closely linked bio-markers to track mechanism. We additionally utilized multi-dosing of BCG combined with frequent monitoring for disease-specific biomarkers for up to 20 weeks to observe any TNF-driven immune effects. Intensive monitoring uncovered alterations in disease-specific T cells and changes in C-peptide secretion that suggest brief functional improvement in the pancreas. Our findings are consistent with trials showing BCG vaccination decreased disease activity and prevented progression of brain lesions in advanced multiple sclerosis, an autoimmune disease similarly sharing autoreactive T cells vulnerable to TNF-triggered cell death [26], [27]. Recent findings also suggest repeat BCG administration, but not single BCG vaccinations in childhood prevents diabetes onset [28] and childhood BCG vaccinations prevent autoantibody formation [20].

In the current study, BCG was expressly chosen as a treatment for its induction of TNF, which has been shown to play a therapeutic role in at least in four rodent models of five autoimmune diseases [3], [7], [8], [10], [12], [29], [30] and *in vitro* [4]. In contrast to the clinical utility of anti-TNF therapies in rheumatoid arthritis but worsening of symptoms when anti-TNF is used in most other autoimmune diseases [31]–[37], these experiments have repeatedly shown that TNF or TNF-inducers protect against onset and progression of many forms of autoimmunity. They also have reversed autoimmune disease, ameliorated advanced autoimmune disease, if administered in newly transplanted islet tissues, and/or permitted regeneration of the end organs. In some of these diverse rodent and human models of autoimmunity, the underlying mechanism of TNF’s therapeutic effect has been traced to various genetic and functional errors in the proteasome or proteasome-activated transcriptional factor NFκB (nuclear factor-κB) signaling pathway [1], [17], [38]–[55].

For a therapeutic and sustained amelioration of the autoimmune state, including a permanent elimination of insulin-autoreactive T cells in diabetes, potentially leading to a sustained return of C-peptide secretion, more frequent or higher dosing of BCG will likely be required. Past human studies have established that even modest levels of remaining C-peptide activity are beneficial in the reduced incidence of retinopathy and nephropathy as well as the avoidance of hypoglycemia [56]. Our findings provide proof-of-principle evidence that insulin-autoreactive T cells can be specifically targeted and eliminated, albeit briefly, *in vivo,* even in long-standing disease with a transient restoration of C-peptide. This paves the way for either higher doses or more frequent BCG administered in future trials for patients with advanced disease to maintain or restore C-peptide levels.

## Supporting Information

Figure S1 **Levels of EBV-specific memory T-cells in placebo subject with latent EBV infection who was not part of this trial** (A) Negative levels of EBV-specific memory T-cells in clinical trial subjects, both BCG-treated and placebo-treated clinical trial subjects.
(TIFF)

Figure S2 **Flow cytometric methods used for the analysis of purified CD8 T-cells for quantifying the numbers of dead versus live cells.** Fresh CD8 T-cells cultured overnight can be demonstrated by forward versus side scatter histograms on a flow cytometer to be either viable or dead based on the placement on a side-scatter versus forward scatter flow gate. The CD8 T cells can additionally be confirmed as dead or alive based not only by the size of dying cells (scatter) but also by staining with propidium iodide (PI), a reagent that stains dead cells. With differential flow gating and/or staining with PI, the dead cells are concentrated in the left upper quadrant and the viable cells are concentrated in the right lower quadrant.
(TIFF)

Checklist S1 **CONSORT Checklist.**
(DOC)

Protocol S1 **Trial Protocol.**
(DOC)
