# Supplementary figures and images for: Proof-of-Concept, Randomized, Controlled Clinical Trial of Bacillus-Calmette-Guerin for Treatment of Long-Term Type 1 Diabetes
Source: PLoS One. 2012 Aug 8;7(8):e41756. doi: 10.1371/journal.pone.0041756 (PMC3414482; doi:10.1371/journal.pone.0041756)

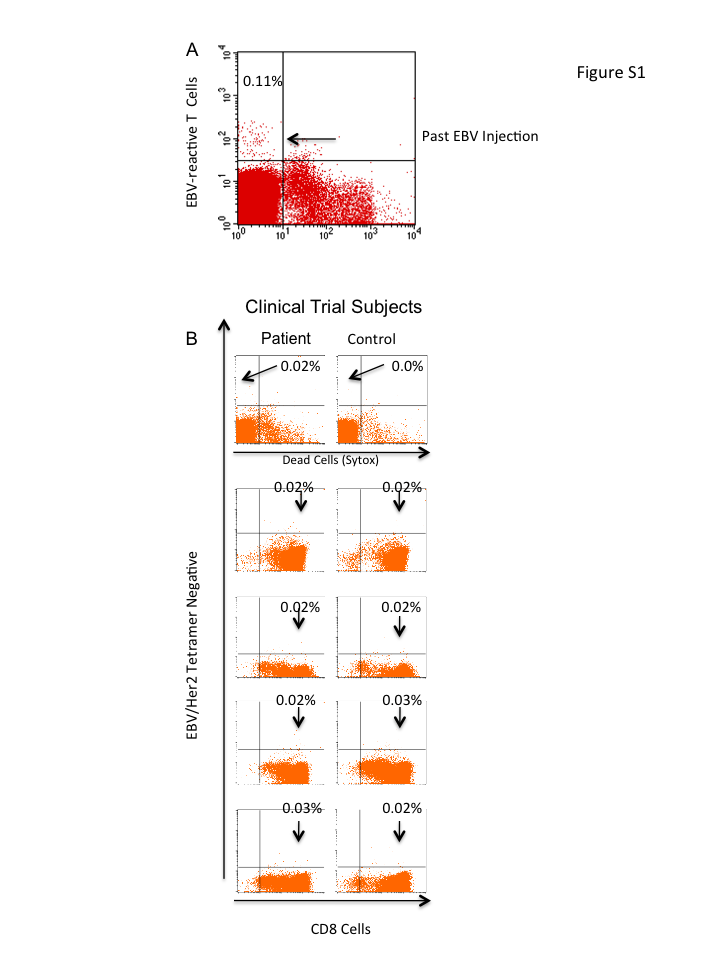

Supplement: Figure S1 — Levels of EBV-specific memory T-cells in placebo subject with latent EBV infection who was not part of this trial (A) Negative levels of EBV-specific memory T-cells in clinical trial subjects, both BCG-treated and placebo-treated clinical trial subjects. (TIFF) [file pone.0041756.s001.tiff]

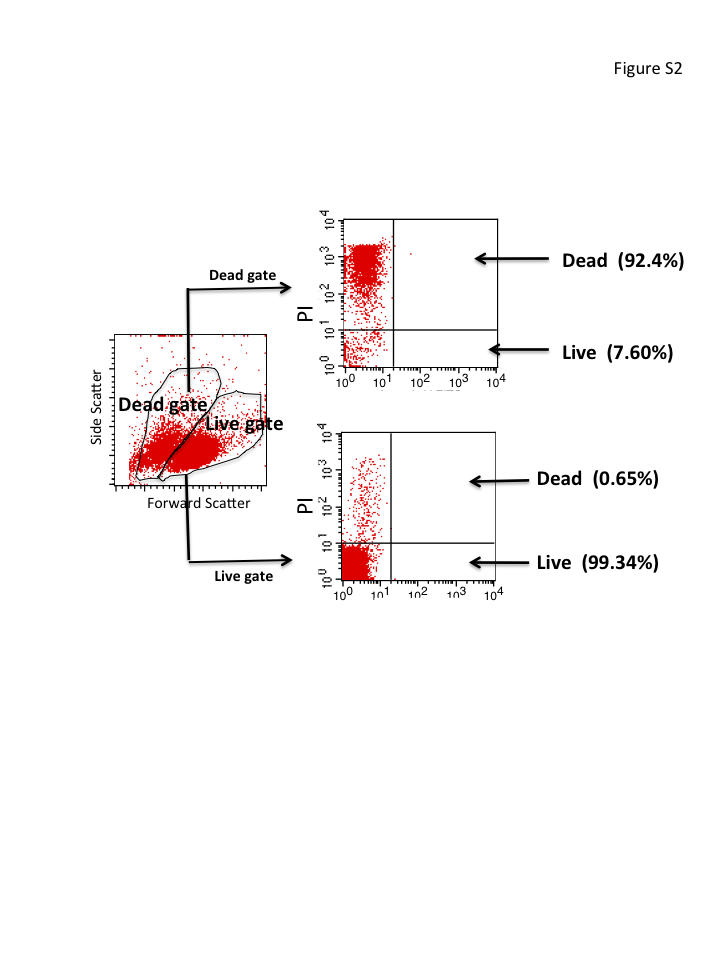

Supplement: Figure S2 — Flow cytometric methods used for the analysis of purified CD8 T-cells for quantifying the numbers of dead versus live cells. Fresh CD8 T-cells cultured overnight can be demonstrated by forward versus side scatter histograms on a flow cytometer to be either viable or dead based on the placement on a side-scatter versus forward scatter flow gate. The CD8 T cells can additionally be confirmed as dead or alive based not only by the size of dying cells (scatter) but also by staining with propidium iodide (PI), a reagent that stains dead cells. With differential flow gating and/or staining with PI, the dead cells are concentrated in the left upper quadrant and the viable cells are concentrated in the right lower quadrant. (TIFF) [file pone.0041756.s002.tiff]
