# Supplementary material for: Proof-of-Concept, Randomized, Controlled Clinical Trial of Bacillus-Calmette-Guerin for Treatment of Long-Term Type 1 Diabetes
Source: PLoS One. 2012 Aug 8;7(8):e41756. doi: 10.1371/journal.pone.0041756 (PMC3414482; doi:10.1371/journal.pone.0041756)
Supplement: Protocol S1 — Trial Protocol. (DOC) [file pone.0041756.s004.doc]

# Answer all questions accurately and completely in order to provide the PHRC with the relevant information to assess the risk-benefit ratio for the study. Do not leave sections blank.

# PRINCIPAL/OVERALL INVESTIGATOR

David M. Nathan, MD

# PROTOCOL TITLE

Determination of Dosing and Frequency of BCG Administration Necessary to Alter T-lymphocyte Profiles in Type 1 Diabetes

# FUNDING

Departmental (Iacocca Foundation), GCRC

# VERSION DATE

July 11, 2008

### SPECIFIC AIMS

Concisely state the objectives of the study and the hypothesis being tested.

To determine whether BCG vaccination can reduce the auto-reactive t-lymphocytes that underlie autoimmunity in the NOD mouse model of Type 1 diabetes and in human Type 1 diabetes. In addition, we will determine whether BCG vaccination increases TNF (tumor necrosis factor, an inflammatory cytokine) levels which underlies the depletion of auto-reactive t-lymphocytes in the NOD mouse. This study is the first step in trying to cure human Type 1 diabetes by altering the underlying autoimmunity.

# BACKGROUND AND SIGNIFICANCE

Provide a brief paragraph summarizing prior experience important for understanding the proposed study and procedures.

Type 1 diabetes is an autoimmune disease mediated by t-lymphocyte destruction of the insulin producing beta-cells of the pancreas. In the NOD mouse model of autoimmune diabetes, which closely resembles human Type 1 diabetes, “poorly educated” auto-reactive t-cells destroy beta-cells. These abnormal lymphocytes (white cells) can be depleted by manipulations that increase TNF. Destroying these abnormal lymphocytes has allowed re-education of the immune system and facilitated beta-cell replacement in the diabetic mice. Moreover, correction of the autoimmunity has resulted in regrowth of islet cells in the pancreas, effectively curing established diabetes in the NOD model of diabetes. If similar results can be obtained in human diabetes, a true cure of this autoimmune disease may be possible. The proposed study is the first step in determining whether manipulations similar to those used in NOD mice are effective in human diabetes.

###### RESEARCH DESIGN AND METHODS

Briefly describe study design and anticipated enrollment, i.e., number of subjects to be enrolled by researchers study-wide and by Partners researchers. Provide a brief summary of the eligibility criteria (for example, age range, gender, medical condition). Include any local site restrictions, for example, “Enrollment at Partners will be limited to adults although the sponsor’s protocol is open to both children and adults.”

In this pilot study, we will randomly assign volunteers with type 1 diabetes to be vaccinated with saline (control group) or with BCG, a bacterial product that has been used to immunize many millions of people against tuberculosis for more than 30 years, to try to deplete the auto-reactive t-lymphocytes in persons with Type 1 diabetes. The BCG will be administered intradermally with the same technique used for vaccination, using the standard vaccination dose. If no untoward side-effects occur, the vaccination will be repeated after a 4 week interval. An increase in TNF levels is the mechanism of auto-reactive t-cell depletion in the NOD mouse model and we will determine whether TNF levels are increased, concurrent with auto-reactive t- lymphocyte depletion, in Type 1 diabetic volunteers after the first and/or second BCG vaccination.

Volunteers, recruited by advertisement or from the MGH Diabetes Clinic, will be asked to come to the Diabetes Research Center. The volunteer will be requested to bring a non-diabetic volunteer. If the type 1 diabetic volunteer cannot identify a non-diabetic volunteer, a separate recruitment will identify such an individual. This non-diabetic subject will provide a blood sample during visits when the diabetic subject has autoreactive T-cells drawn. This will serve as a control sample for the cell-based laboratory analyses. The non-diabetic subject will not have any other interventions. We will recruit volunteers for the study that will be randomized to treatment, placebo or the serially followed reference groups.

Diabetics who are 18 to 55 years old and who have no demonstrable insulin secretion will be eligible. Patients with a history of tuberculosis or a past history of a positive PPD test or previous BCG vaccination, or a history of HIV or chronic skin conditions such as eczema, will be excluded.

The complete Diabetic subject inclusion criteria are:

- Type 1 diabetes treated continuously with insulin from time of diagnosis
- Age 18-55
- Anti-GAD positive
- HIV antibody negative
- Normal CBC
- Negative intermediate PPD test performed and read by study staff
- HCG negative (females)

The complete Diabetic subject exclusion criteria are:

- History of chronic infectious disease, such as HIV
- History of tuberculosis, TB risk factors, or history of + PPD, or BCG vaccination
- Treatment with glucocorticoids (other than intermittent nasal steroids) or disease or condition likely to require steroid therapy
- History of HIV
- Other conditions or treatments associated with increased risk of infections such as patients with a previous history of severe burns, or treatment with immunosuppressive medications of any type (e.g. imuran, methotrexate, cyclosporine, etanercept, infliximab) for any reason
- Current treatment with aspirin > 160 mg/day or chronic, daily NSAIDs
- Fasting or stimulated (1 mg glucagon stimulation test) c-peptide > 0.2 pmol/mL
- History of keloid formation
- HbA1c > 8.0%
- Pregnant or not using acceptable birth control
- Living with someone who is immunosuppressed and/or at high risk for infectious diseases (for example HIV+ or taking immunosuppressive medications for any reason)

Control Non-diabetic Subjects inclusion criteria:

- Age 18-55, no history of autoimmune diseases or diabetes
- No history of HIV
- No history of autoimmune disease or type 1 diabetes (use of insulin continuously since diagnosis) in first degree family members

Briefly describe study procedures. Include any local site restrictions, for example, “Subjects enrolled at Partners will not participate in the pharmacokinetic portion of the study.” Describe study endpoints.

The study will be performed in the outpatient MGH Diabetes Center or in the outpatient General Clinical Research Center. After initial screening, including obtaining baseline blood tests and performing a standard PPD test, diabetic subjects will be randomly assigned to receive either a saline (placebo) or the BCG vaccination. Weekly blood tests will track changes in t-lymphocytes and TNF levels. The BCG vaccination will be repeated 4 weeks after the initial vaccination and will be followed by monitoring for another 16 weeks. Including the screening tests, seventeen visits to the outpatient Diabetes Center or GCRC will be required over the 20-week study. Except for visits three and eight, which will require approximately 3 hours, the visits will take less than 15 minutes.

For studies involving treatment or diagnosis, provide information about standard of care at Partners (e.g., BWH, MGH) and indicate how the study procedures differ from standard care. Provide information on available alternative treatments, procedures, or methods of diagnosis.

STANDARD OF CARE: There is no currently available treatment that can cure or ameliorate the autoimmunity that underlies Type 1 diabetes, once established. Several other currently unapproved, research interventions have been shown to delay the rate of loss of insulin secretion in newly diagnosed patients, but such interventions (e.g. immunosuppressive regimens, autologous bone marrow transplantation) are thought to be ineffective in established diabetes (such as that in the current protocol). Standard therapy of Type 1 diabetes includes use of insulin to achieve glycemic levels as close to the non-diabetic range as possible with the expectation that long-term complications affecting eye, kidney, nerve and cardiovascular system can be reduced, if not eliminated. Such therapy includes frequent (4-6 times per day) monitoring of glucose levels and therapeutic diets. If complications of diabetes develop, they are treated (e.g. laser therapy for eye disease, dialysis or transplantation for end-stage kidney disease). Standard therapy will be continued during this study.

Describe how risks to subjects are minimized, for example, by using procedures which are consistent with sound research design and which do not unnecessarily expose subjects to risk or by using procedures already being performed on the subject for diagnostic or treatment purposes.

MINIMIZATION OF RISK

None of the proposed interventions are routine in the US, although BCG vaccination continues to be used worldwide to protect persons against tuberculosis. Risk from the vaccination, which usually entails the development of an inflammatory papule followed by a scab formation, will be reduced by close, weekly observation of the vaccination site. Any inflammation or scarring more than moderate on an objective scale will result in the repeat vaccination not being given. A small (usually 3-7 mm) scar often develops at the site of vaccination

Describe explicitly the methods for ensuring the safety of subjects. Provide objective criteria for removing a subject from the study, for example, objective criteria for worsening disease/lack of improvement and/or unacceptable adverse events. The inclusion of objective drop criteria is especially important in studies designed with placebo control groups.

ENSURING SUBJECT SAFETY

See minimization of risks above. Any inflammation or scarring associated with the vaccination that is more than moderate on an objective scale will result in the repeat vaccination not being given.

# FORESEEABLE RISKS AND DISCOMFORTS

Provide a brief description of any foreseeable risks and discomforts to subjects. Include those related to drugs/devices/procedures being studied and/or administered/performed solely for research purposes. In addition, include psychosocial risks, and risks related to privacy and confidentiality. When applicable, describe risks to a developing fetus or nursing infant.

The risks entailed in this study are minor and include the minor discomfort and risk of bruising from obtaining blood samples. The total volume of blood obtained over the course of the five-month study will be about 2 times the amount usually given in a single blood donation.

The glucagon stimulated c-peptide test is commonly used in experimental protocols. The glucagon injection may be associated with mild nausea which usually dissipates in 5 minutes. Rarely (less than 1 in 20) subjects may vomit after glucagon.

The tuberculosis test is associated with minimal brief discomfort and, if positive, results in a small lump at the site of the injection.

The saline “vaccination” is associated with minimal, brief discomfort.

BCG vaccinations have been used for more than 30 years in many countries, including Canada and in western Europe, as a vaccination against tuberculosis. The recognized side effects of BCG vaccination include mild local discomfort at the vaccination site with a papular rash developing at the site 10-14 days after vaccination and reaching a maximal diameter of 3 mm 4-6 weeks after vaccination. The rash may scale thereafter, and may leave a visible scar. Local adenopathy is rarely seen in children, but almost never in adults. Rare events include osteomyelitis, lupoid reactions, disseminated BCG infections and death. The frequency of these severe reactions is between 1 in 1,000,000 and 1 in 5,000,000 vaccinations, and have occurred almost exclusively in immunosuppressed children.

Most of the recent experience with BCG has been in the intravesicular treatment of bladder cancer, where weekly instillations of BCG are performed for > 6 weeks.

After BCG vaccination, the tuberculosis skin test (PPD) is no longer reliable and regular chest x-rays may be necessary for specific jobs (teachers, health care workers). Finally, BCG vaccination has been used in Type 1 diabetes without any adverse consequences noted.

**EXPECTED BENEFITS**

# Describe both the expected benefits to individual subjects participating in the research and the importance of the knowledge that may reasonably be expected to result from the study. Provide a brief, realistic summary of potential benefits to subjects, for example, “It is hoped that the treatment will result in a partial reduction in tumor size in at least 25% of the enrolled subjects.” Indicate how the results of the study will benefit future patients with the disease/condition being studied and/or society, e.g., through increased knowledge of human physiology or behavior, improved safety, or technological advances.

BCG vaccination may be protective against tuberculosis, although it is not routinely used in the US. In addition to the general benefit of providing insight into the pathogenesis of Type 1 diabetes and the possible development of a treatment that may result in cure, the patients who participate in this preliminary trial may be eligible for further studies that will determine whether this approach can restore beta cell mass and function, relieving them of their need to administer insulin. The data collected in this phase of the investigation will be applicable in future phases.

**EQUITABLE SELECTION OF SUBJECTS**

The risks and benefits of the research must be fairly distributed among the populations that stand to benefit from it. No group of persons, for example, men, women, pregnant women, children, and minorities, should be categorically excluded from the research without a good scientific or ethical reason to do so. Please provide the basis for concluding that the study population is representative of the population that stands to potentially benefit from this research.

The population selected will represent the majority of persons with Type 1 diabetes who might benefit from reversal of the disease.

When people who do not speak English are excluded from participation in the research, provide the scientific rationale for doing so. Individuals who do not speak English should not be denied participation in research simply because it is inconvenient to translate the consent form in different languages and to have an interpreter present.

Type 1 diabetes, a rare disease, is even more rare in Hispanic populations. That said, the MGH Diabetes Center has investigators and staff who are fluent in Spanish. Potential participants who are Spanish-speaking will not be excluded.

For guidance, refer to the following Partners policy:

Obtaining and Documenting Informed Consent of Subjects who do not Speak English

[**http://healthcare.partners.org/phsirb/nonengco.htm**](http://healthcare.partners.org/phsirb/nonengco.htm)

**RECRUITMENT PROCEDURES**

Explain in detail the specific methodology that will be used to recruit subjects. Specifically address how, when, where and by whom subjects will be identified and approached about participation. Include any specific recruitment methods used to enhance recruitment of women and minorities.

Clinical physicians at the MGH Diabetes Center will discuss the study with Type 1 diabetic patients during their regular visits. If the patients express interest to their physicians, a study coordinator will meet with them and describe the study. Patients who are interested will be provided with the informed consent to take home. Those who are interested will return to the Diabetes Center for a scheduled screening visit during which informed consent will be obtained by the study physician.

We may also utilize advertisements to recruit diabetic and non-diabetic subjects. All advertisements will be submitted to the IRB for approval before use.

Type 1 diabetes mellitus affects men and women equally and we expect approximately 50% women. However, Type 1 diabetes mellitus is relatively rare in minority (African-American and Hispanic) populations, and extremely rare in persons of Asian descent. We will make every effort to identify minority patients with Type 1diabetes and recruit them to the study.

Provide details of remuneration, when applicable. Even when subjects may derive medical benefit from participation, it is often the case that extra hospital visits, meals at the hospital, parking fees or other inconveniences will result in additional out-of-pocket expenses related to study participation. Investigators may wish to consider providing reimbursement for such expenses when funding is available

All expenses related to the study will be covered by the grant, including travel and parking. If research subjects attend visits during mealtimes, incurring possible out of pocket expenses, the study will reimburse them for their meals.

For guidance, refer to the following Partners policies:

Recruitment of Research Subjects

[**http://healthcare.partners.org/phsirb/recruit.htm**](http://healthcare.partners.org/phsirb/recruit.htm)

Guidelines for Advertisements for Recruiting Subjects

[**http://healthcare.partners.org/phsirb/advert.htm**](http://healthcare.partners.org/phsirb/advert.htm)

Remuneration for Research Subjects

[**http://healthcare.partners.org/phsirb/remun.htm**](http://healthcare.partners.org/phsirb/remun.htm)

#### CONSENT PROCEDURES

Explain in detail how, when, where, and by whom consent is obtained, and the timing of consent (i.e., how long subjects will be given to consider participation). For most studies involving more than minimal risk and all studies involving investigational drugs/devices, a licensed physician investigator must obtain informed consent. When subjects are to be enrolled from among the investigators’ own patients, describe how the potential for coercion will be avoided.

The study will be discussed with Type 1 diabetic patients in the MGH Diabetes Center by their clinic physicians during their regular visits. If the patients express interest to their physicians, a study coordinator will meet with them and describe the study. Patients who are interested will be provided with the informed consent to take home. Subjects responding to advertisements will also be provided with a copy of the consent. Those who are interested will return to the Diabetes Center for a scheduled screening visit during which informed consent will be obtained by the study physician.

Potential volunteers will have as long to consider joining the study as necessary. They will be encouraged to bring family members with them to discuss any concerns. An independent Diabetes Investigator-Physician who is not directly involved with the current study will obtain informed consent from any patients of the Principal Investigator who express interest in joining the study in order to ensure that overt or subtle coercion has not played a role in their volunteering for the study.

NOTE: When subjects are unable to give consent due to age (minors) or impaired decision-making capacity, complete the forms for Research Involving Children as Subjects of Research and/or Research Involving Individuals with Impaired Decision-making Capacity, available on the New Submissions page on the PHRC website:

**[http://healthcare.partners.org/phsirb/newapp.htm#Newapp](http://healthcare.partners.org/phsirb/newapp.htm#Newapp)**

For guidance, refer to the following Partners policy:

Informed Consent of Research Subjects

[**http://healthcare.partners.org/phsirb/infcons.htm**](http://healthcare.partners.org/phsirb/infcons.htm)

## DATA AND SAFETY MONITORING

Describe the plan for monitoring the data to ensure the safety of subjects. The plan should include a brief description of (1) the safety and/or efficacy data that will be reviewed; (2) the planned frequency of review; and (3) who will be responsible for this review and for determining whether the research should be altered or stopped. Include a brief description of any stopping rules for the study, when appropriate. Depending upon the risk, size and complexity of the study, the investigator, an expert group, an independent Data and Safety Monitoring Board (DSMB) or others might be assigned primary responsibility for this monitoring activity.

NOTE: Regardless of data and safety monitoring plans by the sponsor or others, the principal investigator is ultimately responsible for protecting the rights, safety, and welfare of subjects under his/her care.

An independent Data Safety Monitoring Board, with expertise in clinical immunology and diabetes, will be formed and will be charged with overseeing the study for safety and efficacy. Any severe adverse events, as well as a summary of all vaccine related events, will be reported to the DSMB in parallel with reporting such events to the HRC and FDA. Absent such events, the progress of the study with regard to adverse events and outcomes (results of T-cell measures and cytokine measurements, in particular) will be reviewed with the DSMB after the first 6 subject pairs (3 diabetics treated with the active vaccine) have completed the study.

Describe the plan to be followed by the Principal Investigator/study staff for review of adverse events experienced by subjects under his/her care, and when applicable, for review of sponsor safety reports and DSMB reports. Describe the plan for reporting adverse events to the sponsor and the Partners’ IRB and, when applicable, for submitting sponsor safety reports and DSMB reports to the Partners’ IRBs. When the investigator is also the sponsor of the IND/IDE, include the plan for reporting of adverse events to the FDA and, when applicable, to investigators at other sites.

NOTE: In addition to the adverse event reporting requirements of the sponsor, the principal investigator must follow the Partners Human Research Committee guidelines for Adverse Event Reporting

See above

## MONITORING AND QUALITY ASSURANCE

Describe the plan to be followed by the principal investigator/study staff to monitor and assure the validity and integrity of the data and adherence to the IRB-approved protocol. Specify who will be responsible for monitoring, and the planned frequency of monitoring. For example, specify who will review the accuracy and completeness of case report form entries, source documents, and informed consent.

NOTE: Regardless of monitoring plans by the sponsor or others, the principal investigator is ultimately responsible for ensuring that the study is conducted at his/her investigative site in accordance with the IRB-approved protocol, and applicable regulations and requirements of the IRB.

The PI and study coordinator will be responsible for overseeing the quality assurance regarding the conduct of the study.

Adherence to the protocol, including strict adherence to the eligibility criteria for recruitment, oversight of vaccinations and safety monitoring, and collection of data will be examined on a regular basis, need for corrective actions noted, and follow-up to determine that any identified problems are rectified will be the responsibility of the PI. Any outside (non-MGH) laboratory, including commercial laboratories, will be reviewed to ensure that quality control is at a level that is consistent with clinical research.

The PI will present a summary of the monitoring to the DSMB on a regular basis, and no less frequently than every 6 months.

For guidance, refer to the following Partners policies:

##### Data and Safety Monitoring Plans and Quality Assurance

[**http://healthcare.partners.org/phsirb/datasafe.htm**](http://healthcare.partners.org/phsirb/datasafe.htm)

Adverse Event Reporting Guidelines

[**http://healthcare.partners.org/phsirb/adverse.htm**](http://healthcare.partners.org/phsirb/adverse.htm)

# PRIVACY AND CONFIDENTIALITY

Describe methods used to protect the privacy of subjects and maintain confidentiality of data collected. This typically includes such practices as substituting codes for names and/or medical record numbers; removing face sheets or other identifiers from completed surveys/questionnaires; proper disposal of printed computer data; limited access to study data; use of password-protected computer databases; training for research staff on the importance of confidentiality of data, and storing research records in a secure location.

NOTE: Additional measures, such as obtaining a Certificate of Confidentiality, should be considered and are strongly encouraged when the research involves the collection of sensitive data, such as sexual, criminal or illegal behaviors.

Privacy will be protected by assigning the volunteers unique identifiers that will be kept securely (in a locked cabinet) in the Diabetes Center. No one outside of study staff will have access to the identifiers, which will not be able to be tracked to personal identifiers. Moreover, HIPAA regulations will be strictly observed.

SENDING SPECIMENS/DATA TO RESEARCH COLLABORATORS OUTSIDE PARTNERS

Specimens or data collected by Partners investigators will be sent to research collaborators outside Partners, indicate to whom specimens/data will be sent, what information will be sent, and whether the specimens/data will contain identifiers that could be used by the outside collaborators to link the specimens/data to individual subjects.

Outside laboratories may be used to analyze the research samples for selected analytes. The outside laboratories will not have access to any data (personal identifiers) that could be used to identify the volunteers. A unique study identifier will be attached to such specimens with the key matching the subject to the sample kept securely by the PI at the MGH Diabetes Center.

Specifically address whether specimens/data will be stored at collaborating sites outside Partners for future use not described in the protocol. Include whether subjects can withdraw their specimens/data, and how they would do so. When appropriate, submit documentation of IRB approval from the recipient institution.

Not applicable

# RECEIVING SPECIMENS/DATA FROM RESEARCH COLLABORATORS OUTSIDE PARTNERS

When specimens or data collected by research collaborators outside Partners will be sent to Partners investigators, indicate from where the specimens/data will be obtained and whether the specimens/data will contain identifiers that could be used by Partners investigators to link the specimens/data to individual subjects. When appropriate, submit documentation of IRB approval and a copy of the IRB-approved consent form from the institution where the specimens/data were collected.

N/A
